# Region-by-region analysis of PET, MRI, and histology in en bloc-resected oligodendrogliomas reveals intra-tumoral heterogeneity

**DOI:** 10.1007/s00259-018-4107-z

**Published:** 2018-08-14

**Authors:** Kenney Roy Roodakker, Ali Alhuseinalkhudhur, Mohammed Al-Jaff, Maria Georganaki, Maria Zetterling, Shala G. Berntsson, Torsten Danfors, Robin Strand, Per-Henrik Edqvist, Anna Dimberg, Elna-Marie Larsson, Anja Smits

**Affiliations:** 1 Department of Neuroscience, Neurology, Uppsala University, University Hospital, S-751 85 Uppsala, Sweden; 2 Department of Surgical Sciences, Radiology, Uppsala University, Uppsala, Sweden; 3 Department of Information Technology, Division of Visual Information and Interaction, Uppsala University, Uppsala, Sweden; 4 Department of Immunology, Genetics and Pathology, Rudbeck Laboratory, Uppsala University, Uppsala, Sweden; 5 Department of Neuroscience, Section of Neurosurgery, Uppsala University, Uppsala, Sweden; 6 Department of Immunology, Genetics and Pathology and Science for Life Laboratory, Uppsala University, Uppsala, Sweden; 7 Department of Radiology, Uppsala University Hospital, Uppsala, Sweden; 8 Institute of Neuroscience and Physiology, Department of Clinical Neuroscience, Sahlgrenska Academy, University of Gothenburg, Gothenburg, Sweden

**Keywords:** Perfusion MR, ^11^C-methionine PET, Proliferation, Vascularization, Co-registration

## Abstract

**Purpose:**

Oligodendrogliomas are heterogeneous tumors in terms of imaging appearance, and a deeper understanding of the histopathological tumor characteristics in correlation to imaging parameters is needed. We used PET-to-MRI-to-histology co-registration with the aim of studying intra-tumoral ^11^C-methionine (MET) uptake in relation to tumor perfusion and the protein expression of histological cell markers in corresponding areas.

**Methods:**

Consecutive histological sections of four tumors covering the entire en bloc-removed tumor were immunostained with antibodies against IDH1-mutated protein (tumor cells), Ki67 (proliferating cells), and CD34 (blood vessels). Software was developed for anatomical landmarks-based co-registration of subsequent histological images, which were overlaid on corresponding MET PET scans and MRI perfusion maps. Regions of interest (ROIs) on PET were selected throughout the entire tumor volume, covering hot spot areas, areas adjacent to hot spots, and tumor borders with infiltrating zone. Tumor-to-normal tissue (T/N) ratios of MET uptake and mean relative cerebral blood volume (rCBV) were measured in the ROIs and protein expression of histological cell markers was quantified in corresponding regions. Statistical correlations were calculated between MET uptake, rCBV, and quantified protein expression.

**Results:**

A total of 84 ROIs were selected in four oligodendrogliomas. A significant correlation (*p* < 0.05) between MET uptake and tumor cell density was demonstrated in all tumors separately. In two tumors, MET correlated with the density of proliferating cells and vessel cell density. There were no significant correlations between MET uptake and rCBV, and between rCBV and histological cell markers.

**Conclusions:**

The MET uptake in hot spots, outside hotspots, and in infiltrating tumor edges unanimously reflects tumor cell density. The correlation between MET uptake and vessel density and density of proliferating cells is less stringent in infiltrating tumor edges and is probably more susceptible to artifacts caused by larger blood vessels surrounding the tumor. Although based on a limited number of samples, this study provides histological proof for MET as an indicator of tumor cell density and for the lack of statistically significant correlations between rCBV and histological cell markers in oligodendrogliomas.

**Electronic supplementary material:**

The online version of this article (10.1007/s00259-018-4107-z) contains supplementary material, which is available to authorized users.

## Introduction

Oligodendrogliomas are glial tumors believed to originate from oligodendrocytes, and are further classified based on molecular tumor features [1]. Classical oligodendrogliomas are characterized by isocitrate dehydrogenase (IDH) gene mutations and codeletions on chromosome 1p/19q. In the absence of diagnostic molecular testing, oligodendrogliomas are classified as oligodendrogliomas NOS (not-otherwise-specified), while IDH mutated non-codeleted gliomas are classified as astrocytomas [2]. Oligodendrogliomas are most common around the age of 35–44 [3]. Current median survival time for patients with oligodendrogliomas WHO grade III is 12–14 years, and longer for patients with oligodendroglioma WHO grade II [2]. Primary treatment is maximal safe resection, followed by radiotherapy and chemotherapy for high-risk patients [4, 5]. Based on their chemosensitivity, it has recently been suggested that chemotherapy may be the optimal primary treatment for oligodendrogliomas [6].

The histopathological diagnosis of oligodendrogliomas is based on the presence of neoplastic cells with morphological characteristics resembling oligodendrocytes. Oligodendroglial tumors are highly cellular lesions consisting of relatively small but densely packed tumor cells [2, 7, 8]. The tumor periphery consists of less cellular areas but with diffuse infiltrative growth and clustering of tumor cells around the perikarya of preexistent neurons, under the pial surface, and surrounding small cortical vessels [2]. Oligodendrogliomas are further characterized by branching networks of capillaries (chicken-wire pattern) that set them apart from astrocytomas.

Contrast-enhanced magnetic resonance imaging (MRI) is widespread available and routinely used for radiological tumor diagnosis, showing superior soft-tissue resolution. Key features in oligodendrogliomas are the presence of calcifications and cortical thickening with typical involvement of both the cortex and the subcortical white matter [9]. Oligodendroglial tumors show and a more solid, infiltrative, or mixed growth pattern [10]. In general, the differentiation of heterogeneous glioma tissue from surrounding edema on MRI is unreliable [11], and MRI tends to underestimate the actual tumor extent of oligodendrogliomas [10].

Advanced MRI methods such as perfusion-weighted imaging (PWI) and PET with amino acid tracers provide complementary diagnostic information in gliomas [12, 13]. In case histopathological diagnosis is based on partial tumor resection or biopsy with subsequent risk for misclassification and undergrading [14], amino acid PET provides important additional diagnostic accuracy since hot spot areas generally represent the most malignant tumor part [15, 16]. Studies using serial stereotactic biopsies have confirmed the superiority of PET with ^11^C-methionine (MET) over CT or MRI to delineate the tumor [17, 18]. During recent years, ^18^F-fluoroethyl-L-tyrosine (FET) has received increased attention and MET has been replaced by FET for the clinical management of gliomas in many centers due to the logistic advantages of FET [12, 19]. FET PET-MRI-guided diagnostic biopsy showed a specificity of 53% for MRI alone versus 94% for combined PET-MRI [20].

The uptake of amino acid tracers is known to reflect the transport mediated by amino acid carriers over the endothelial cell membrane, and to correlate with the microvessel density of the tumor [21]. However, few studies have directly compared the amino acid uptake with histological tumor features. All studies so far have been based on multiple biopsies or partial tumor resections and have focused on single hot spot regions in the tumor [20, 21]. As for today, a deeper understanding of how intra-tumoral variations in tracer uptake and tumor perfusion correlate with specific histological features of the tumor is lacking. It remains unclear whether the tracer uptake measured by PET, or tumor perfusion measured by PWI, outside hot spot areas and in the tumor periphery is a measure for cellular proliferation, tumor cell density, or vessel density. This information is valuable in a number of clinical situations where PET and advanced MRI are part of the diagnostic work-up for patients with gliomas, such as radiotherapy planning, evaluation of response to therapy, and detection of recurrence [12, 21]. We have recently described a new method for co-registration of MR images and histological images in en bloc surgically removed gliomas, allowing region-by-region comparisons of the entire tumor with infiltrating tumor edge [22]. Here, we further developed our technique to perform region-by-region correlations between PET, MRI perfusion and histological images. Our aim was to provide an exact correlation between changes in MET uptake, tumor perfusion, and histological tumor characteristics of oligodendrogliomas, covering the entire tumor volume, including the infiltrating tumor edge.

## Materials and methods

### Tumor samples

**Fig. 1:**
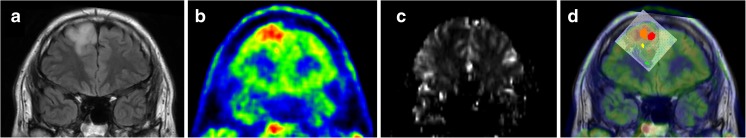
Patient 1. **a** T2-weighted FLAIR MRI shows a hyperintense slightly heterogeneous tumor in the right frontal lobe.** b** MET PET shows the hotspot region of the tumor.** c** DSC perfusion MRI with rCBV greyscale map shows predominantly low or normal perfusion in the region corresponding to the PET hotspot.** d** Co-registration of MRI, PET and corresponding histological images with defined ROIs (ROI_1_,* red* and* orange*; ROI_2_* yellow*, ROI_3_* green*)

**Fig. 2:**
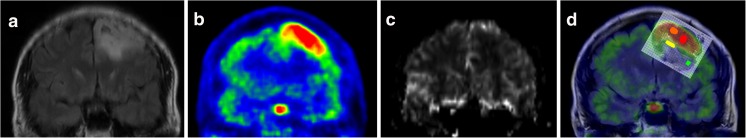
Patient 2.** a** T2-weighted FLAIR MRI shows a hyperintense tumor with small cystic regions in the left frontal lobe.** b** MET PET shows the hotspot region of the tumor.** c** DSC perfusion MRI with rCBV greyscale map shows higher perfusion in the region corresponding to the PET hotspot.** d** Co-registration of MRI, PET, and corresponding histological images with defined ROIs (ROI_1_,* red* and* orange*; ROI_2_* yellow*, ROI_3_* green*)

**Fig. 3:**
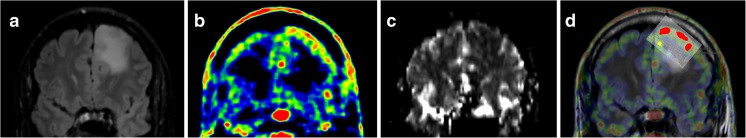
Patient 3.** a** T2-weighted FLAIR MRI shows a hyperintense tumor in the left frontal lobe.** b** MET PET shows the hotspot regions of the tumor.** c** DSC perfusion MRI with rCBV greyscale map shows low or normal perfusion in the region corresponding to the PET hotspot.** d** Co-registration of MRI, PET, and corresponding histological images with defined ROIs (ROI_1_,* red* and* orange*; ROI_2_* yellow*). There was no identifiable ROI_3_ in this sequence

**Fig. 4:**
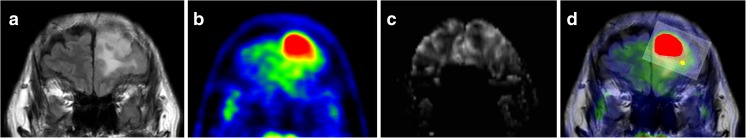
Patient 4.** a** T2-weighted FLAIR MRI shows a left frontal hyperintense tumor with minimal contrast enhancement on T1-weighted images (not shown).** b** MET PET shows a large hotspot region in the tumor.** c** DSC perfusion MRI with rCBV greyscale map shows areas with low, normal, and high perfusion in the region corresponding to the PET hotspot.** d** Co-registration of MRI, PET, and corresponding histological images with defined ROIs (ROI_1_,* red* and* orange*; ROI_2_* yellow*). There was no identifiable ROI_3_ in this sequence. As shown, ROI_1_ covers almost the entire tumor volume

In our recently published co-registration study, we included five patients with suspected diffuse low-grade glioma (DLGG) that were suitable for en bloc tumor resection [22]. Four of these five patients had histopathological diagnosis of oligodendrogliomas (Figs. 1, 2, 3, and 4) and were included in the present study. The institutional review board approved the protocol for the present analysis, and written informed consent was obtained prior to patient participation. A summary of the clinical characteristics of these patients and histopathological diagnoses is shown in Table 1.

**Table 1:** Individual data for the four patients with oligodendrogliomas WHO grade II–III

Patient no.	Age	Sex	Histological tumor type	WHO grade	Localization	Tumor volume on FLAIR (ml)	Postop cavity (ml)	Tissue weight (g)	1p/19q
1	33	Male	Oligodendroglioma	II	Frontal pole, R	28	58	67	Co-del
2	50	Female	Oligodendroglioma	II	Frontal pole, L	45	56	38	Co-del
3	39	Male	Oligodendroglioma	II	Frontal pole, L	68	83	87	non Co-del
4	53	Male	Oligodendroglioma	III	Frontal pole, L	84	85	82	Co-del

*R* right,* L* left,* ml* milliliter,* g* grams,* Co-del* co-deleted

### PET and MRI acquisition

MRI was performed according to our original study protocol [23], using a 3-Tesla scanner (Achieva, Philips Healthcare, Best, The Netherlands), including perfusion sequences. Morphological imaging included axial and coronal T2-weighted fluid attenuated inversion recovery (FLAIR) scans (0.45 × 0.45 × 6 mm^3^ voxel size). Dynamic susceptibility contrast (DSC) perfusion imaging was performed as previously described [24], with a T2*W single-shot gradient echo-EPI sequence (1.7 × 2.3 × 5.0 mm^3^). Whole-brain coverage was obtained using these parameters.

PET scanning was performed on a Discovery ST PET/CT (General Electric Medical Systems) in a 3D acquisition mode. This scanner has 24 detector rings with 420 detectors resulting in 47 image planes with a plane separation of 3.27 mm and a total axial field of view of 157 mm. Images were reconstructed using ordered-subsets expectation maximation (OSEM; 2 iterations, 15 subsets, 128 × 128 matrix) with a 2.14 mm FWHM post-filter applying appropriate corrections such as scatter, randoms, and attenuation correction based on a low-dose CT scan. Voxel size of the resulting images was 1.95 × 1.95 × 3.27 mm and the spatial resolution 6 mm. The tracer ^11^C-Methionine (4.0 MBq/kg) was injected 25 min before start. Scan time was 10 min, 1 frame.

PET and MRI were performed at a mean of 34 days (range, 20-52 days) respectively 28 days (range, 5-53 days) before surgery.

### Tissue preparation and immunohistochemistry

**Fig. 5:**
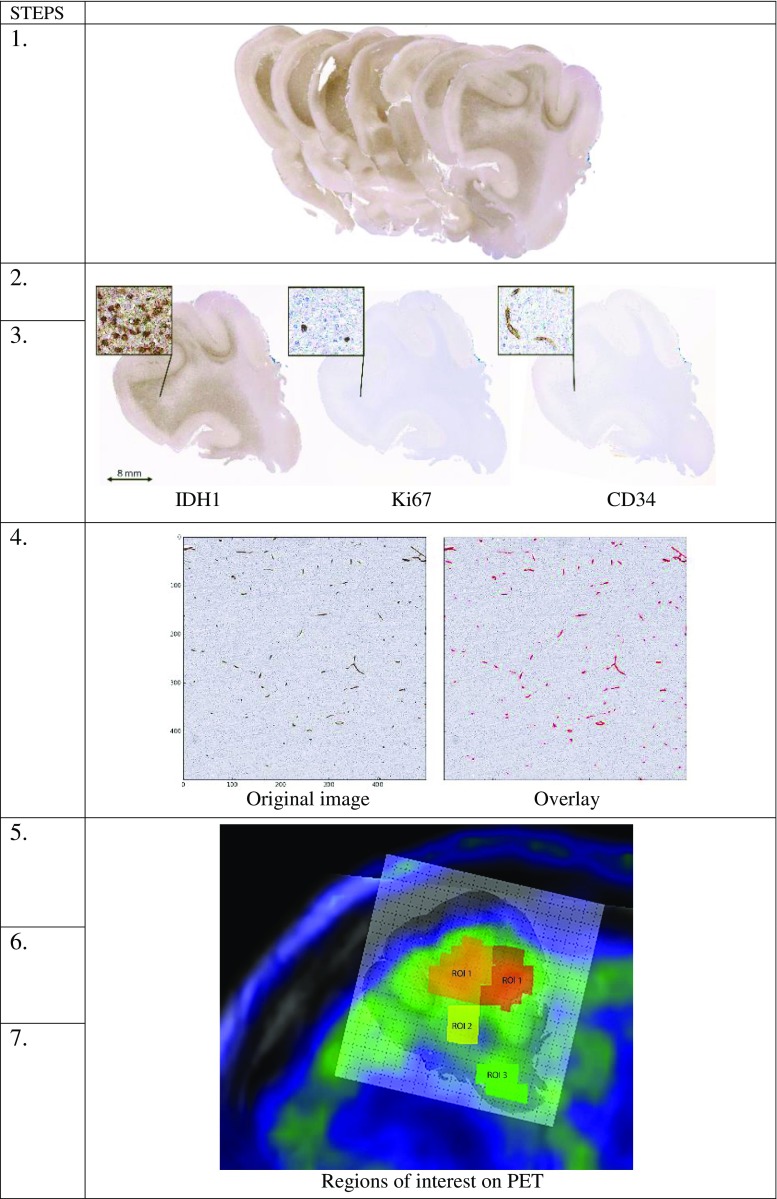
Illustration of the study design. Step 1: En bloc tumor resection of 8–12 tissue slices of 6–8 mm thickness. Steps 2–3: Development of software for anatomical landmark-based co-registration of consecutive microsections (4 μm) for immunohistochemical staining of for tumor cells (IDH1), proliferating cells (Ki67), and blood vessels (CD34). Step 4: Quantification of protein expression in each histological sub-image (500 × 500 pixels). The original image of CD34 protein expression (*left*) was overlaid with the identified objects (*red*) in CellProfiler. Steps 5–7: Manual co-registration of MRI, PET, perfusion maps and corresponding histological images. Selection of ROIs on PET covering hot spot areas (ROI_1_), areas outside hot spots (ROI_2_) and tumor periphery (ROI_3_). Analysis of correlations between MET uptake and quantified protein expression, MET uptake and rCBV, and rCBV and quantified protein expression. Note: ROIs in the figures indicate their specific location but not their exact volume

Figure 5 illustrates the design of the study with the different steps that have been performed. Preparation of the en bloc tumor tissues was performed as previously described [22]. After resection, the tumor surfaces (anterior, posterior, medial, and lateral) were color-marked for spatial orientation. The en bloc tissue sample was fixed in formalin, subsequently cut into consecutive tissue slices, again fixed in formalin, and embedded in paraffin wax. Serial microsections were rehydrated prior to immunohistochemical staining with antibodies against the tumor cell marker IDH1-mutated protein (IDH1-R132H), the proliferation marker Ki67 and the hematopoietic progenitor cell antigen CD34 (Fig. 5, steps 1–2).

### Co-registration of consecutive histological images

Software was developed for anatomical landmark-based co-registration of consecutive histological images of various immunostainings. In summary, a landmark-based multi-step elastic image registration was designed using an in-house Matlab application (The MathWorks, Inc., Natick, MA, USA) and implemented allowing a region-by-region comparison of histological assessment [25]. A set of 5–10 anatomical landmarks was found accurate enough for consecutive co-registration of serial histological images (Fig. 5, step 3). Each histological image was divided into sub-images (500 × 500 pixels) for region-by-region quantification of protein expression of histological cell markers, using an additional in-house Matlab application (see Supplementary file for software description).

### Quantification of immunohistochemical stainings

Automated region-by-region analysis of protein expression was performed using an in-house built pipeline in the CellProfiler software [26] (see Supplementary file). We created a pipeline of individual modules that were able to estimate localization, intensity, shape, and size of protein expression for the three histological cell markers (IDH1-mutated protein as a marker for tumor cells, Ki67 as a marker for proliferating cells and CD34 as a marker for blood vessels). Each separate sub-image (500 × 500 pixels) traveled through the pipeline and was processed by each module to quantify the absolute count per square of protein expression. In the final analysis, mean values of protein expression were calculated in each region of interest defined on MET PET (see under PET analysis). This allowed region-by-region comparison between MET uptake, tumor perfusion, and protein expression (Fig. 5, step 4).

### Co-registration of histology, PET, and MRI

As a first step, we imported original co-registrations of MRI and histological images (denoted as *co-registration 1*) created in our previous study [22] into Carimas software (kindly provided by the Turku PET center, Finland). We then imported the T2-FLAIR images that were co-registered with the *co-registration 1* image using the Normalized Mutual Information (NMI) tool by Carimas [27, 28]. Following this step, we imported both MET PET scans and MRI perfusion maps (cerebral blood volume, CBV) into the project and co-registered these to the FLAIR images using the same NMI tool (denoted as *co-registration 2*). This way, all co-registered images had similar orientation (Fig. 5, step 5).

### PET analysis

Analysis of MET PET was performed using Carimas 2.9 software (Turku PET center, Finland). MET uptake in regions of interest (ROIs), defined as described below, was calculated as tumor-to-normal tissue (T/N) ratio, i.e., the ratio between the maximum standardized uptake value (SUV), reflecting the value of the pixel with highest radioactivity in the ROIs and the mean uptake in the contralateral normal brain. The normal tissue reference region was drawn as a 1-cm-thick cortical ROI at the level of the basal ganglia, ranging from the occipital to the frontal cortex in the contralateral hemisphere. Additionally, we defined a deep white matter reference region in the contralateral hemisphere above and lateral to the ventricle.

Serial ROIs were systematically selected on all MET PET scans. We defined three different types of ROIs based on MET uptake in the entire tumor volume: ROI_1_ = areas with highest MET uptake (hotspot), ROI_2_ = areas with medium MET uptake, and ROI_3_ = areas with lower MET uptake located in the infiltrating tumor border (Fig. 5, step 6).

### MRI perfusion analysis

DSC perfusion was analyzed as previously described [24], with calculation of CBV maps using nordicICE software (NordicNeuroLab AS, Bergen, Norway). Relative CBV (rCBV) was calculated as tumor-to-normal tissue (T/N) ratio by dividing the mean CBV in ROIs defined on PET, with the mean CBV within a reference area located in normal appearing white matter of the contralateral hemisphere above and lateral to the ventricle.

### Pre-processing and area definition

To compare quantified protein expression of histological cell markers within the ROIs defined on PET, we applied a grid using Adobe Photoshop CC (Adobe Systems Software). The grid was placed on top of *co-registration 2*, enabling a detailed and direct comparison between radiological and histological images. All histological sub-images (500 × 500 pixels) within one specific ROI were selected using the grid. This procedure was applied for each histological image of all four tumors. In the final analysis, we used the mean count of protein expression per ROI for each of the three histological cell markers (Fig. 5, step 7)*.*

### Statistical analysis

Statistical analysis was performed for the whole resection in each patient separate. The correlation between different variables (MET, rCBV, IDH1 count, Ki67 count, CD34 count) within the ROIs for each tumor was analyzed using non-linear correlation (Spearman rho; two-tailed *p* values with 95% confidence intervals) in JMP software (version 13).

## Results

We made an effort to systematically identify the three types of ROIs (ROI_1–3_) in all PET scans (Figs. 1, 2, 3, and 4). Thus, 84 representative ROIs were selected in four tumors (47 ROI_1_, 21 ROI_2_, 16 ROI_3_). These 84 ROIs covered a total number of 2134 histological sub-images (size, 500 × 500 pixels) in which protein expression of three histological cell markers (IDH1, Ki67, and CD34) was quantified. Due to technical reasons, a number of immunostainings within the 84 selected ROIs were missing or not suitable for quantification (31 missing for Ki67; 15 missing for CD34). In the final analysis, the following parameters were included: MET (*n* = 84), rCBV (*n* = 84), IDH1 counts (*n* = 84), Ki67 counts (*n* = 53) and CD34 counts (*n* = 69). The results for each tumor are described below, followed by a summary of the data.

### Patient 1

A 33-year-old man had a non-enhancing heterogeneous tumor in the right frontal pole on MRI. En bloc resection was performed as described previously [22] and histopathological examination showed an oligodendroglioma WHO grade II with IDH1 mutation, 1p/19q codeletion, and Ki67 < 5% (Table 1). A total of 29 ROIs were selected on PET; 16 areas with highest MET uptake (ROI_1_), seven areas with medium uptake (ROI_2_), and six areas with lower uptake in the tumor periphery (ROI_3_) (Fig. 1).

The MET uptake and rCBV values and the quantified protein counts (mean number of counts per ROI) in all 29 ROIs, as well as the statistical correlations between the various parameters are presented in Table 2. As shown, there was a strong correlation between MET uptake and tumor cell density (MET-IDH1: *r* = 0.91; *p* < 0.0001), MET uptake and vessel density (MET-CD34: *r* = 0.73; p < 0.0001), and MET uptake and proliferation (MET-Ki67: *r* = 0.71; *p* = 0.0465) in this tumor. No significant correlation was found between MET uptake and tumor perfusion (MET-rCBV: *r* = 0.06; *p* = 0.74). In addition, rCBV showed no significant correlations with histological cell markers.

**Table 2:** MET uptake, rCBV values, and counts of histological cell markers, with statistical correlations between the various parameters for all four patients

Patient 1	MET	rCBV	IDH1	Ki67	CD34
ROI 1 (16)	1.58	1.10	1741.59	59.71	273.32
ROI 2 (7)	1.06	1.10	454.92	32.94	133.49
ROI 3 (6)	0.80	0.90	36.66	16.07	108.98
MET (29)	N/A	*r* = 0.06; *p* = 0.7384	*r* = 0.91; *p* < 0.0001*	*r* = 0.71; *p* = 0.0465*	*r* = 0.73; *p* < 0.0001*
rCBV (29)	*r* = 0.06; *p* = 0.7384	N/A	*r* = 0.06; *p* = 0.7481	*r* = 0.07; *p* = 0.8665	*r* = 0.23; *p* = 0.2309
Patient 2	MET	rCBV	IDH1	Ki67	CD34
ROI 1 (8)	2.43	1.56	3214.15	88.44	353.49
ROI 2 (4)	1.38	0.80	3278.65	95.81	343.79
ROI 3 (5)	0.88	1.14	343.44	162.04	409.31
MET (17)	N/A	*r* = 0.36; *p* = 0.1524	*r* = 0.51; *p* = 0.0345*	*r* = 0.15; *p* = 0.5604	*r* = 0.43; *p* = 0.3965
rCBV (17)	*r* = 0.36; *p* = 0.1524	N/A	*r* = 0.10; *p* = 0.6942	*r* = 0.24; *p* = 0.3531	*r* = 0.20; *p* = 0.7040
Patient 3	MET	rCBV	IDH1	Ki67	CD34
ROI 1 (14)	1.22	1.66	976.89	168.18	340.70
ROI 2 (5)	0.84	1.32	758.37	75.55	136.01
ROI 3 (4)	0.63	0.85	341.07	54.24	114.17
MET (23)	N/A	*r* = 0.28; *p* = 0.1912	*r* = 0.44; *p* = 0.0371*	*r* = 0.69; *p* = 0.0095*	*r* = 0.67; *p* = 0.0005*
rCBV (23)	*r* = 0.28; *p* = 0.1912	N/A	*r* = 0.10; *p* = 0.6343	*r* = 0.53; *p* = 0.0640	*r* = 0.35; *p* = 0.0977
Patient 4	MET	rCBV	IDH1	Ki67	CD34
ROI 1 (9)	2.79	2.02	68.73	183.14	371.61
ROI 2 (5)	1.43	1.63	3.13	178.33	215.74
ROI 3 (1)	0.85	2.35	0.22	180.89	637.20
MET (15)	N/A	*r* = 0.08; *p* = 0.7710	*r* = 0.85; *p* < 0.0001*	*r* = 0.25; *p* = 0.3688	*r* = 0.02; *p* = 0.9496
rCBV (15)	*r* = 0.08; *p* = 0.7710	N/A	*r* = 0.15; *p* = 0.5848	*r* = 0.16; *p* = 0.5585	*r* = 0.49; *p* = 0.0664

### Patient 2

A 50-year-old woman was diagnosed with a tumor in the left frontal pole with heterogeneous signal characteristics on MRI (Fig. 2). The tumor was removed en bloc and histopathological examination showed oligodendroglioma WHO grade II with densely packed IDH1-labeled tumor cells located mainly in the grey matter [22]. Molecular analysis showed IDH1 mutation, Ki67 < 5% and 1p/19q codeletion (Table 1). A total of 17 ROIs were selected of which eight representing regions with highest MET uptake (ROI_1_), four with medium uptake (ROI_2_), and five with lower uptake located in the tumor periphery (ROI_3_). The MET uptake, rCBV value, and protein expression of histological markers in these ROIs are presented in Table 2. There was a statistically significant correlation between MET uptake and IDH1 count (MET-IDH1: *r* = 0.51; *p* = 0.0345). As shown in Table 2, there were no significant correlations between MET uptake with tumor perfusion and with expression of Ki67 and CD34. We observed that several of the ROI_3_ in this tumor were located adjacent to or partially overlapping with the cortex, with inherent higher perfusion (Table 2).

### Patient 3

A 39-year-old man was examined showing a non-enhancing, slightly heterogeneous tumor and en bloc tumor resection was performed. Histopathological examination showed a WHO grade II glial tumor with exclusively oligodendrocytic differentiation. Molecular analysis showed IDH1 mutation but no 1p19q codeletion. In spite of the intact 1p19q chromosomes, the tumor was morphologically diagnosed as an oligodendroglioma based on its characteristic oligodendroglial phenotype throughout the entire resection (Table 1). A total of 23 ROIs were selected, of which 14 in hot spot regions (ROI_1_), five in areas with medium uptake (ROI_2_), and four in areas with lower uptake in the tumor periphery (ROI_3_) (Fig. 3) (Table 2). There was a significant correlation between MET uptake and tumor cell count (MET-IDH1: *r* = 0.44; *p* = 0.0371), proliferation count (MET-Ki67: *r* = 0.69; *p* = 0.0095), and vessel count (MET-CD34: *r* = 0.67; *p* = 0.0005). No significant correlations were present between tumor perfusion and histological cell markers.

### Patient 4

A 53-year-old man was diagnosed with a left frontal tumor showing minimal contrast enhancement on MRI (Fig. 4). En bloc resection was performed, with some loss of white matter tissue on the medial/inferior side of the tumor. Histopathological examination showed IDH1-mutated codeleted oligodendroglioma WHO grade III, Ki67 proliferation rate was 25% (Table 1) [22]. A total of 15 ROIs were identified on PET, of which nine in the hot spot (ROI_1_), five with medium uptake (ROI_2_), and due to loss of white matter tissue during en bloc resection only one representative ROI_3_ with lower MET uptake located in the tumor periphery. Statistical analysis showed a strong correlation between MET uptake and IDH1 (MET-IDH1: *r* = 0.85; *p* < 0.0001) (Table 2). No significant correlation was found between MET uptake and tumor perfusion or the expression of other histological markers. Similar to patient 2, we observed that the single ROI_3_ in this tumor was located adjacent to the cortex, resulting in inherent increased perfusion values.

### Summary of findings

MET uptake was consistently correlated with tumor cell density throughout the entire tumor volume in all four oligodendrogliomas. Similar results were obtained using white matter as reference area for MET uptake (data not shown). Tumor perfusion in the ROIs defined by MET uptake did not correlate with MET uptake or with any of the histological cell markers.

In two tumors (patients 1 and 3), MET uptake also correlated with the density of proliferating cells and blood vessels. These two tumors showed consistently decreasing rCBV values at longer distance from the tumor core. In the other tumors (patients 2 and 4), we noticed an unexpected increase of rCBV values in ROI_3_, which was due to partial volume effects with adjacent cortex and large vessels in the peritumoral region.

## Discussion

In the present study, we performed region-by-region comparisons of MET uptake, tumor perfusion, and protein expression of histological cell markers in a series of en bloc-resected oligodendrogliomas. It has been shown that the uptake of amino acid tracers is an indirect measure of microvessel density [29], but studies correlating uptake with proliferation, tumor cell density, and vessel density have shown incomplete or partly contradicting results [30–37]. With the aim to complement previous reports and to provide the proof-of-concept of how MET uptake and rCBV reflect the cellular heterogeneity in oligodendrogliomas, we evaluated the entire volume in en bloc-resected oligodendrogliomas. Our main finding is that MET unanimously reflects the density of tumor cells. By using the antibody against IDH1-mutated protein as a marker for tumor cells, we confirm previously established correlations between MET and cell density, and extend these findings to a correlation between MET and tumor cell density in oligodendrogliomas [21].

In addition, we found a significant correlation between MET uptake and microvessel density in two of four oligodendrogliomas, one codeleted and one non-codeleted tumor. Several studies have indicated that the generally higher MET uptake in oligodendrogliomas compared to astrocytomas is related to higher cell density, but also to higher microvessel density in these tumors [21, 38]. Indeed, MET uptake has been correlated with microvessel density in oligodendrogliomas independent of malignancy grade [29]. Our results confirm previous findings and show that MET uptake is a measure for microvessel density also in regions outside the hot spot and in peritumoral areas. In this context, the lack of significant correlations between MET-CD34 and MET-Ki67 in two tumors (patient 2 and patient 4) needs further explanation. In one tumor (case 4), we were able to include just one single ROI_3_ representing tumor periphery, due to minor loss of peritumoral tissue during the en bloc resection. In the other tumor (patient 2), several ROI_3_ were located adjacent to or partially overlapping with the cortex, with inherent higher perfusion. Selected ROIs were defined on PET and not on perfusion maps, since hot spot identification on rCBV maps in non-enhancing gliomas is challenging with high inter-observer variability [39, 40]. Therefore, we could not control for this artifact. It is likely though that the ROIs_3_ with increased count for microvessels and proliferating cells are not true histological representations of the tumor periphery, explaining the lack of statistically significant correlations between MET-CD34 and MET-Ki67 in these tumors.

Expression of the Ki67 antigen used as a proliferation marker is present in cycling cells [41], and correlates with malignancy in gliomas [42]. The PET literature regarding MET and FET uptake and proliferation in gliomas is not conclusive [20, 30–33, 43]. Fractions of cycling tumor cells in the tumor can be found at the tumor center as well as near the inner limits of the MRI-defined abnormalities, which may explain some of the inconsistencies between studies [44]. In the present study, we used absolute values of Ki67 count, instead of the proliferation ratio between cycling cells and tumor cells. The significant MET-Ki67 correlations in two of four oligodendrogliomas are consistent with previous reports and exemplify that the relationship between MET uptake and intra-tumoral cell proliferation in oligodendrogliomas is not unambiguous. One would expect that it is more difficult to establish statistically significant correlations between MET uptake and proliferation in low-grade tumors with low Ki67 index (< 5%). However, this was not the case. Significant MET-Ki67 correlations were found in low-grade oligodendrogliomas but not in the anaplastic oligodendroglioma with Ki67 index of 25%.

The development and maintenance of an adequate blood supply by cell proliferation and angiogenesis are essential for tumor growth and invasion [37, 45, 46]. Previous reports have shown that the rCBV measured by MRI perfusion is a measure for cell density [37], tumor proliferation [47], and vascularization [48] in glioma. In this study, we did not find any significant correlations between rCBV and the markers for tumor cell density, proliferation, or vessel cell density. It should be reminded, however, that the ROIs used for quantifying rCBV values were based on MET PET and not on perfusion maps. Our particular study design might also explain the lack of significant correlation between MET uptake and rCBV. Overall, we clearly demonstrate that MET PET has advantages over PWI, which is in line with previous studies [49, 50], but in contrast to others [51].

In conclusion, we further developed a previously described co-registration of histological and radiological images for region-by-region comparison of MET uptake, tumor perfusion, and histological cell markers in en bloc removed oligodendroglioma. Our key finding is the strong correlation between MET uptake and tumor cell density, in the entire tumor volume as well as the infiltrating border. MET uptake is correlated to the density of microvessels and proliferating cells as well, but this correlation is somewhat less stringent and probably more susceptible to measurement artifacts in regions covering the tumor periphery. The novelty of the present study lies in the use of en bloc-resected tumors, representing the concept of supra-total tumor resections. Previous studies have all been based on limited, less representative tumor tissue [52]. Our model also illustrates a novel way of evaluating imaging parameters with respect to the intra-tumoral heterogeneity of gliomas.

### Limitations of the study

One limitation of this study is the small number of patients, which is however inherent to the methodological design of the study. Only a selection of gliomas can be surgically removed by en bloc tumor resection. It should also be noted that we applied four separate analyses of intra-tumoral heterogeneity, which means that comprehensive data were not pooled for inter-tumoral analysis. Instead, we analyzed multiple ROIs in each tumor and aimed to identify common patterns between tumors. Future studies with larger tumor samples and including astrocytic tumor subtypes are needed to confirm our data. In addition, there are some technical limitations to be considered. Both MET uptake and rCBV were measured in vivo before surgery and compared with ex vivo markers. Therefore, anesthesia or surgery may have affected microvessel contractility [48]. We encountered no significant difficulties during fusion of conventional MRI and PET images using the Carimas software. The Normalized Mutual Information (NMI) tool used in this study has previously proven to be highly accurate, with only a 1.2% failure [28]. In our previous work, we provided a detailed description of the limitations occurring during the manual process of histology-to-radiology co-registration [22]. Deformations and shrinkage that occur when tissue is processed for histology (i.e., formalin fixation, sectioning, and staining) make the procedure of co-registration challenging. Potential mismatch between PET/MRI and histological parameters has been minimized by applying a grid to the en bloc tumor resections to select specific areas, and by calculating mean values within relatively large ROIs to account for inaccuracies. However, the selection of ROIs located adjacent to or partially overlapping the cortex has probably influenced our results. Finally, a fixed pipeline was performed in CellProfiler to prevent bias, but not all histological stainings were done simultaneously, which could cause batch-related variations with regard to relative immunohistochemical staining intensities.

## Electronic supplementary material

ESM 1 (DOCX 18 kb)
