# Supplementary material for: Region-by-region analysis of PET, MRI, and histology in en bloc-resected oligodendrogliomas reveals intra-tumoral heterogeneity
Source: Eur J Nucl Med Mol Imaging. 2018 Aug 14;46(3):569–79. doi: 10.1007/s00259-018-4107-z (PMC6351509; doi:10.1007/s00259-018-4107-z)
Supplement: Supplementary file 1 — (DOCX 18 kb) [file 259_2018_4107_MOESM1_ESM.docx]

SUPPLEMENTARY MATERIAL

**Region-by-region analysis of PET, MRI and histology in *en bloc* resected oligodendrogliomas reveals intra-tumoral heterogeneity**

Kenney Roy Roodakker*, Ali Alhuseinalkhudhur, Mohammed Jaff, Maria Georganaki, Maria Zetterlin5, Shala G Berntsson, Torsten Danfors, Robin Strand, Per-Henrik Edqvist, Anna Dimberg, Elna-Marie Larsson, Anja Smits.

**Description of the used software**

*ManualCPR*

We developed a custom in-house built software called ManualCPR, written in Matlab^®^. The software enables co-registration of two images interactively through user-specified control points (anatomical landmarks). The user is required to load/input two images, a 'fixed' image (F) and a moving image (M), with the fixed image acting as the reference to which the moving image is to be co-registered on. The co-registration is initiated by selecting a minimal number of 5 control points (landmarks in both F and M, corresponding to similar anatomical locations). Once the control points are chosen, built-in Matlab functions are used to compute a projective transformation matrix by a least-squares optimization approach with the control-points as constraints. The computed transformation matrix is then applied to M to get a preliminary registered image M' that the user sees alongside F and M. In an additional sub-window of the graphical user interface, options are available to display the difference image (F-M') as well as the overlay of M' on F for optimal visual evaluation of the quality of the registration, i.e. M' relative to F. Once M' has been obtained, ManualCPR lets the user to respectively 1) Tweak the registration by going back and adding more/less/different control-points and generating a new M', and 2) Further transform M' by using M' as the moving image (i.e. M'=M) and performing control-point select step, and 3) Finally save M' in various file formats when the user is satisfied with the registration.

*Chopper*

We developed a custom in-house built software called Chopper, written in Matlab^®^, that functions as an image sectioning tool. After image load, Chopper overlays NxN pixel squares in a checkerboard fashion, extracts and saves each square in a directory named as the original loaded image where N is specified by the user.

*Cellprofiler*

We created a pipeline using the Cellprofiler^®^ software. An images module was designed and names were assigned to each image. Images were grouped into image subsets, based on histological cell markers, to be independently processed independently. A three-step strategy-based module was created for IDH1-mutated protein expression containing image processing (color to gray scale conversion), object identification/segmentation (applying thresholds) and measurement (object count and area occupied). Likewise, a 3-step module was created for Ki67 and CD34 for image processing (color deconvolution), object segmentation, and measurement (object count and area occupied). The accuracy of final quantification was examined by overlaying the identified objects on top of the original image in CellProfiler.
